# Vest’s Eulogy on the Dog

**Published:** 1906-11

**Authors:** 


### Vest's Eulogy on the Dog.

One of the most famous speeches ever made by the late Senator Vest, of Missouri, was made in the course of the trial of a man who had wantonly shot a dog belonging to a neighbor. Vest represented the plaintiff, who demanded $200 damages. When Vest finished speaking, the jury, after two minutes' deliberation, awarded the plaintiff $500. The full text of the speech is printed below:

"Gentlemen of the Jury: The best friend a man has in this world may turn against him and become his enemy. His son or daughter that he has reared with loving care may prove ungrateful. Those who are nearest and dearest to us, those whom we trust with our happiness and our good name, may become traitors to their faith. The money that a man has he may lose; flies away from him, perhaps when he needs it most. A man's reputation may be sacrificed in a moment of ill-considered action. The people who are prone to fall on their knees to do us honor when success is with us, may be first to throw the stone of malice when failure settles its cloud upon our heads. The one absolutely unselfish friend that man can have in this selfish world, the one that never deserts him, the one that never proves ungrateful or treacherous, is the dog.

"Gentlemen of the jury, a man's dog stands by him in prosperity and in poverty, in health and in sickness. He will sleep on the cold ground, where the wintry winds blow and the snow drives fiercely, if only he may be near his master's side. He will kiss the hand that has no food to offer, he will lick the wounds and sores that come in encounter with the roughness of the world. He guards the sleep of his pauper master as if he were a prince. When all other friends desert he remains. When riches take wings and reputation falls to pieces, he is as constant in his loyalty as the sun in its journey through the heavens. If fortune drives the master forth an outcast in the world, friendless and homeless, the faithful dog asks no higher privilege than that of accompanying him to guard against danger, to fight against his enemies; and when the last scene of all comes, and death holds the master in its embrace, and his body is laid away in the cold ground, no matter if all other friends pursue their way, there by his graveside will the noble dog be found, his head between his paws, his eyes sad but open, in alert watchfulness, faithful and true even to death."

---

SUPPRESSION of urine in infants is extremely rare, and in any case in which the child is unable to pass its urine it is far more likely that there is some congenital source of obstruction. The existence of this should be determined by the prompt introduction of a soft rubber catheter.—*International Journal of Surgery.*

---

IN CASES of spina bifida in which the mass is small and the overlying skin not much atrophied, the protruding part may be reduced into the vertebral canal and prevented from returning by a disc of pasteboard, cork, or celluloid, held in position by adhesive plaster and bandage.—*International Journal of Surgery.*

